# Deletion of a Genetic Region of lp17 Affects Plasmid Copy Number in *Borrelia burgdorferi*

**DOI:** 10.3389/fcimb.2022.884171

**Published:** 2022-04-12

**Authors:** Jessica K. Wong, Michael A. Crowley, Troy Bankhead

**Affiliations:** Department of Veterinary Microbiology and Pathology, Washington State University, Pullman, WA, United States

**Keywords:** *Borrelia burgdorferi*, Lyme disease, LP17, bbd21, paralogous family 32, plasmid copy number control

## Abstract

*Borrelia burgdorferi*, the Lyme disease pathogen, is maintained in its enzootic life cycle through complex gene regulatory pathways encoded on its uniquely fragmented genome. This genome consists of over 20 plasmids, and the regulatory mechanisms of plasmid maintenance and replication are largely unknown. The *bbd21* gene, encoded on lp17 and a member of the paralogous family 32 proteins, was originally proposed to be a putative *parA* orthologue involved with plasmid partitioning; however, this function has not been confirmed to date. To determine the role of *bbd21* in *B. burgdorferi*, we utilized targeted gene deletion and discovered *bbd21* and *bbd22* are co-transcribed. The effects of *bbd21* and *bbd22* deletion on plasmid copy number and mammalian infectivity were assessed. By qPCR, lp17 copy number did not differ amongst strains during mid-exponential and stationary growth phases. However, after *in vitro* passaging, the mutant strain demonstrated an 8-fold increase in lp17 copies, suggesting a cumulative defect in plasmid copy number regulation. Additionally, we compared lp17 copy number between *in vitro* and mammalian host-adapted conditions. Our findings showed 1) lp17 copy number was significantly different between these growth conditions for both the wild type and *bbd21-bbd22* deletion mutant and 2) under mammalian host-adapted cultivation, the absence of *bbd21-bbd22* resulted in significantly decreased copies of lp17. Murine infection studies using culture and qPCR demonstrated *bbd21*-*bbd22* deletion resulted in a tissue colonization defect, particularly in the heart. Lastly, we showed *bbd21* transcription appears to be independent of direct *rpoS* regulation based on similar expression levels in wild type and Δ*rpoS*. Altogether, our findings indicate the *bbd21*-*bbd22* genetic region is involved with regulation of lp17 plasmid copy number. Furthermore, we propose the possibility that lp17 plasmid copy number is important for microbial pathogenesis by the Lyme disease spirochete.

## Introduction

Lyme disease is the most prevalent vector-borne illness in North America with over 30,000 infections (Kugeler et al., 2021) and a health-related cost of $786M USD (Mac et al., 2019) reported each year in the United States alone. The causative agent, *Borrelia burgdorferi*, survives in an enzootic life cycle that requires a tick and a mammalian host. The bacterium has adapted to this unique lifestyle through the evolution of a small, complex genome composed of a single linear chromosome and up to 22 circular and linear plasmids, depending on the strain (Barbour and Garon, 1987; Barbour, 1988; Ferdows and Barbour, 1989; Stevenson et al., 1996; Casjens et al., 1997; Fraser et al., 1997; Casjens et al., 2000; Iyer et al., 2003). Generally, genes encoded on the chromosome are involved with housekeeping functions such as DNA replication, transcription, translation, solute transport, and metabolism (Casjens et al., 1997; Fraser et al., 1997), while those on plasmids encode virulence factors considered important for host adaptation (Coburn et al., 2021). Global expression of these genes is responsive to environmental cues and known to be regulated by three pathways: the BosR/Rrp2/RpoN/RpoS alternative sigma (σ) factor cascade, the Hk1/Rrp1 two-component system and c-di-GMP, and the stringent response mediated by RelBbu and DksA (Samuels et al., 2022). Linear versus circular plasmid topology has also been shown to play a factor in gene expression (Beaurepaire and Chaconas, 2007). While regulation of plasmid gene expression has been well-studied, less is known regarding regulation of plasmid maintenance and replication.

Typically, bacterial plasmid replication is tightly regulated by fixed copy numbers and permitted growth under specific conditions to achieve stable coexistence of plasmids and minimization of metabolic requirements (del Solar and Espinosa, 2000). In *B. burgdorferi*, plasmids are similarly reported to be maintained in low copy number of approximately one per chromosome (Hinnebusch and Barbour, 1992; Beaurepaire and Chaconas, 2007), in which there is an estimated one per cell (Morrison et al., 1999). All *Borrelia* plasmids have a set of related putative plasmid maintenance genes (Dunn et al., 1994; Zückert and Meyer, 1996; Fraser et al., 1997; Casjens et al., 2000). These genes are usually arranged in clusters and can confer autonomous replication (Stewart et al., 2001; Eggers et al., 2002; Stewart et al., 2003; Byram et al., 2004; Beaurepaire and Chaconas, 2005; Dulebohn et al., 2011). The genes encode five paralogous families (PFs) of proteins designated PF32, PF49, PF50, PF57, and PF62 (Fraser et al., 1997; Casjens et al., 2000). The roles of PF49 and PF50 are unknown. PF57 and PF62 are grouped together based on their limited homology and similar function as replication initiators (Stewart et al., 2001; Eggers et al., 2002; Stewart et al., 2003; Beaurepaire and Chaconas, 2005).

Except for lp5 and cp9, a PF32 member is present on all *Borrelia* plasmids (Casjens et al., 2000), signifying their importance to spirochete survival. The original study on these maintenance genes reported that PF32 is the only *Borrelia* PF with sequence homology to known DNA maintenance proteins in the ParA superfamily, including ParA involved with plasmid partitioning in *Escherichia coli*, Soj involved with chromosome segregation in *Bacillus subtilis*, and RepB involved with plasmid copy number control in *Enterococcus faecalis* (Zückert and Meyer, 1996). Interestingly, subsequent studies have proposed the *bbd21* gene residing on the lp17 plasmid as a putative *parA* orthologue solely to the ParA protein of *E.coli* phage P1 (Casjens et al., 2000; Beaurepaire and Chaconas, 2005; Deneke and Chaconas, 2008). The *bbd21* locus (NC_001849.2) is composed of a 741 bp open reading frame (ORF) that overlaps by 10 bp with *bbd22*, a 270 bp ORF (NC_001849.2), and the genes are oriented in the same direction. While *bbd22* is predicted as a hypothetical protein with unknown function, there is evidence to support *bbd21* as a putative *parA* orthologue, including a ~25% sequence homology with the ParA family and presence of a Walker box motif characteristic of the ParA proteins (Zückert and Meyer, 1996; Casjens et al., 2000). Additionally, *bbd21* has demonstrated ATPase activity required for segregation of plasmids in other bacteria (Deneke and Chaconas, 2008).

Despite this body of evidence, confirmation that *bbd21* acts as a *parA* orthologue with partitioning function is lacking. ParA requires ParB, a DNA binding adaptor protein, to function in plasmid partitioning (Sengupta and Austin, 2011), yet a *parB* orthologue has not been described in the *Borrelia* genome to date. This is not surprising, given that the ParB proteins are considered diverse, and sequence homology can be uncommon (Gerdes et al., 2000). Nonetheless, when *bbd21* was deleted along with a large segment of lp17 in the noninfectious B31A strain, there were no reported aberrations in plasmid copy number, plasmid stability, or plasmid incompatibility (Beaurepaire and Chaconas, 2005). Previous work from our laboratory likewise found that deletion of a similar region in the infectious B31-5A4 strain did not result in changes to lp17 stability (Casselli et al., 2012). Another study reported multiple transposon insertions in *bbd21*, suggesting the gene is not required for lp17 replication and maintenance (Lin et al., 2012). Others have also found the PF32 member on cp26 to be dispensable (Tilly et al., 2012). One proposed alternative function to plasmid partitioning in the PF32 genes includes preventing plasmid incompatibility within the cp32 plasmids (Eggers et al., 2002). Another alternative function involves plasmid replication based on physical interaction of recombinant *bbd14* and *bbd21*, although additional experiments in the same study did not support this proposition (Deneke and Chaconas, 2008). These aforementioned studies were limited by qualitative gel electrophoresis techniques, which lacks sensitivity to subtle quantifiable changes in plasmid copy number, replication, and incompatibility. Moreover, in the most recent article on this topic, authors of some of the previous studies casted doubt on the function of *bbd21* as a *parA* orthologue, describing the designation as “premature and potentially inaccurate” (Chaconas and Norris, 2013). As a result, the role of *bbd21* in *B. burgdorferi* remains unknown. Given the high retention of lp17 in *B. burgdorferi* clinical isolates and during cultivation (Purser and Norris, 2000; Casjens et al., 2018), and the increasing evidence that lp17 serves as a regulatory hub that modulates expression of virulence factors important for host infection (Sarkar et al., 2011; Casselli et al., 2012; Casselli et al., 2019; Medina-Pérez et al., 2020), it is critical to elucidate the mechanisms of lp17 plasmid regulation in order to understand disease pathogenesis and identify potential targets for Lyme disease prevention and therapeutic intervention.

To investigate the role of *bbd21*, we generated a targeted gene deletion mutant and studied the effect on plasmid copy number and mammalian infectivity. We demonstrate co-transcription with *bbd22*, alterations to lp17 copy number *in vitro* and in mammalian host-adapted spirochetes, and impaired mammalian tissue colonization. Our findings indicate the *bbd21-bbd22* region is involved with regulation of lp17 plasmid copy number, and furthermore, suggest plasmid copy number is important for microbial infection and tissue colonization by the Lyme disease spirochete.

## Materials and Methods

### Ethics Statement

The experiments on mice and rats were carried out according to the protocols and guidelines approved by the American Association for Accreditation of Laboratory Animal Care (AAALAC) and by the Office of the Campus Veterinarian at Washington State University (Animal Welfare Assurance A3485-01 and USDA registration number 91-R-002). These guidelines are in compliance with the U.S. Public Health Service Policy on Humane Care and Use of Laboratory Animals. The animals were housed and maintained in an AAALAC-accredited facility at Washington State University, Pullman, WA. The Washington State University Institutional Animal Care and Use Committee approved the experimental procedures carried out during the current studies (Animal Safety Approval Forms # 6760 and 6701).

### Bacterial Strains and Culture Conditions

Detailed descriptions of the *B. burgdorferi* strains used in these studies are presented in **Supplementary Table 1**. The *B. burgdorferi* isolate B31-5A4 (5A4) was kindly provided by George Chaconas and the 5A4 Δ*rpoS* mutant was kindly provided by Melissa Caimano. The B31 strain has been sequenced (Fraser et al., 1997; Casjens et al., 2000) and the infectivity and plasmid profiles have been previously described (Purser and Norris, 2000). *B. burgdorferi* were grown at 35°C and 1.5% CO_2_ in modified BSK-II (Pollack et al., 1993) supplemented with 6% rabbit serum (Cedarlane Laboratories). Mutant strains were grown with appropriate antibiotics, including kanamycin (200 µg/ml), gentamicin (100 µg/ml), or streptomycin (100 µg/ml). *Borrelia* cultures were passaged no more than three times prior to use in experiments. Cell densities and growth phase were monitored by dark-field microscopy and enumerated using a Petroff-Hausser counting chamber.

*E. coli* strains were grown in Luria broth (LB) or LB agar with appropriate antibiotics, including kanamycin (50µg/ml) or gentamicin (5µg/ml).

### Plasmid Construction

The deletion mutant construct was generated using the general protocol described in (Magunda and Bankhead, 2016). Primers are shown in **Table S2**. Because *bbd21* and *bbd22* open reading frames (ORFs) on lp17 are transcribed in the same direction and overlap by 10 bp, a targeted internal gene deletion approach of *bbd21* was utilized to minimize disruption to *bbd22* and its promoter region. The internal deletion was designed 260 bp upstream of the *bbd22* start codon to avoid putative promoter sequences. The lp17 target region of 1195 bp including the *bbd21* ORF and a 305 bp upstream and a 149 bp downstream flanking region was PCR amplified from wild-type 5A4 genomic DNA with primers P933 and P934. The amplicon was cloned into the vector pCR-Blunt-II-TOPO (Invitrogen) and maintained in One Shot Stbl3 cells (Invitrogen). The internal deletion was generated by PCR amplification with primers P996 and P997 of this plasmid to exclude a 408 bp region within the *bbd21* ORF and to insert AgeI and PacI restriction sites. The gentamicin-resistance cassette (*gent*) driven by a *flgB* promoter was PCR amplified with primers P998 and P508 from pPH25 (Hove et al., 2014) with insertion of AgeI and PacI restriction sites. The amplicon was cloned into the vector pCR-Blunt-II TOPO (Invitrogen) and propagated in Top10 cells (Invitrogen). Following restriction enzyme digest with AgeI and PacI, *flgB-gent* was ligated into the deleted region of *bbd21*, generating the plasmid pJW16. The construct was maintained in EC19 cells (Hove et al., 2014) as EC276. DNA was isolated using a plasmid midikit (Sigma). Successful cloning was verified for correct size and orientation by restriction enzyme digest and sequencing before transformation into *B. burgdorferi* competent cells.

### Mutant Generation and Confirmation

All transformations were performed using electrocompetent *B. burgdorferi* prepared as previously described (Bankhead and Chaconas, 2007; Samuels et al., 2018). *E. coli* DNA was isolated using a plasmid midikit (Sigma), and *B. burgdorferi* DNA was isolated using a plasmid maxikit (Sigma). The deletion mutant strain, which was found to lack expression of both *bbd21* and *bbd22* (Δ*21-22*) was generated by allelic exchange following electroporation of 50 µg of DNA isolated from EC276 and transformed into the 5A4 wild-type strain. After numerous unsuccessful attempts to complement *bbd21* back into its native locus in the deletion mutant, a previously published alternative strategy using whole plasmid replacement (Grimm et al., 2004; Imai et al., 2013; Raffel et al., 2014) was utilized to generate the complement strain, lp17comp. A 5A4 lp17 mutant in which *bbd01-bbd03* were replaced with a kanamycin resistance cassette (*kan*) by telomere resolution was utilized (Tourand et al., 2003; Casselli et al., 2019). This strain was previously reported to not affect murine infectivity or tissue colonization (Casselli et al., 2019). Approximately 18 µg of plasmid DNA was electroporated into Δ*21-22* electrocompetent cells. Mutants were initially screened by PCR followed by Southern blot analyses for the respective presence or absence of the internally deleted portion of *bbd21*, *gent*, and *kan* (primers shown in **Supplementary Table 2**). Endogenous plasmid content was identified by multiplex PCR using primers previously described (Bunikis et al., 2011).

### Southern Blot Hybridization

*B. burgdorferi* genomic DNA was isolated by phenol-chloroform extraction. Genomic DNA was separated using 0.65% agarose gel electrophoresis at 80V for 18 h with pulse field inversion and recirculating buffer followed by bidirectional transfer to two nylon membranes (Roche). Probes for *bbd12-bbd13* of lp17, the internal deletion of *bbd21*, *gent*, and *kan* were generated from plasmid template DNA using primers indicated in **Table S2** with the DIG Probe Synthesis kit (Roche), per manufacturer’s instructions. Bands were detected with the DIG Luminescent Detection kit (Roche).

### *In Vitro* Growth Assays

Growth assays were performed as previously described (Casselli et al., 2012). Briefly, *B. burgdorferi* strains were grown to late log phase and subcultured to 10^5^ cells/ml. Cell densities were determined at 24 h intervals for 9 days. Values were expressed as mean density ± standard deviation from three experiments.

### *In Vitro* Passaging

A protocol similar to (Grimm et al., 2003) was followed for *in vitro* passaging of *B. burgdorferi*. Briefly, wild type, Δ*21-22*, and lp17comp were inoculated from frozen glycerol stocks and grown in parallel in 5 ml cultures of liquid BSK II medium with appropriate antibiotics to late exponential phase (5 x 10^7^ to 1 x 10^8^ cells/ml) and then passaged 1:500 into fresh medium 25 times. Final cultures were grown to a density of 8 x 10^7^ to 1.0 x 10^8^ cells/ml. All cultures were pelleted and stored at -20°C.

### lp17 Plasmid Copy Number

To investigate lp17 plasmid copy number over the growth curve, genomic DNA was extracted from aliquots of 10 ml cultures of wild-type, Δ*21-22*, and lp17comp strains grown to mid-exponential phase (1.8 x 10^7^ to 2.6 x 10^7^ cells/ml) and then stationary phase (1.1 x 10^8^ to 1.3 x 10^8^ cells/ml). Next, the effect of multiple passages on lp17 plasmid copy number was studied. Genomic DNA was extracted from strains passaged 25 times and was compared to controls (P1), which were the same strains cultivated directly from glycerol stocks that were passaged no more than three times prior to the start of experiments. To determine the effect of different growth environments on plasmid copy number, mammalian-host adapted wild-type, Δ*21-22*, and lp17comp spirochetes were compared to *in vitro*-cultivated spirochetes. qPCR for *bbd14* and *flaB* (primers and probes listed in **Table S2**) was performed in duplex with three technical replicates per sample using TaqMan probes and the QX200 Droplet Digital PCR (ddPCR) system (Bio-Rad Laboratories), including the QX200 Droplet Generator, the PX1 PCR Plate Sealer, and the QX200 Droplet Reader, all per manufacturer’s instructions. PCR was performed on the C1000 Touch Thermal Cycler (Bio-Rad Laboratories) using the following cycling conditions: enzyme activation at 95°C for 10 min, amplification for 40 cycles with a ramp rate of 2.5°C/s for denaturation at 94°C for 30 s and annealing and extension at 60°C for 1 min, enzyme deactivation at 98°C for 10 min, and an optional hold at 4°C. Values were expressed as relative copies of *bbd14* per *flaB* and calculated as an average ± standard error mean.

### Mammalian Host-Adapted Spirochetes

Mammalian host-adapted spirochetes were generated by cultivation in dialysis membrane chambers (DMCs) implanted into the peritoneal cavity of rats, as previously described (Caimano, 2018). The following modifications and specifications to the DMC protocol were applied. Sprague-Dawley rats (Envigo, Livermore, CA) were male, 7-8 weeks old, and weighed 220 – 250 g. Sterile surgical instruments included a new disposable No. 10 scalpel blade used for each animal, and an instrument pack, including 13.3 cm Mayo-Hegar needle holders, 11 cm iris scissors, 14 cm rat tooth forceps, and 14 cm Adson forceps, used aseptically on up to 3 animals. Instead of ketamine/xylazine for anesthesia, rats were maintained on isoflurane gas (1-3%; VetOne). In addition to perioperative and postoperative carprofen, rats were subcutaneously administered preoperative carprofen (5 mg/kg, VetOne) 24 h prior to surgery and perioperative buprenorphine SR (1 mg/kg, ZooPharm LLC), as well as local lidocaine (5 mg/kg, VetOne) injected as a line block along the planned incision site. Perioperative fluids (LRS or saline, 3-5% body weight) were administered to support hydration, and animal body temperature was maintained with a heating pad during surgery prep and surgery. Instead of wound clips, the skin incision was closed with a Precise Multi-Shot MS Disposable Skin Stapler (3M) and liquid skin adhesive. Each DMC contained 12 ml of diluted culture. All DMCs were explanted on day 14 post surgery.

### RT-PCR/qRT-PCR

For confirmation of functional gene deletion in the Δ*21-22* mutant and assessment of *rpoS* regulation of *bbd21*, three 5ml cultures of each *Borrelia* strain were grown to late log phase (8 x 10^7^ to 1 x 10^8^ cells/ml), and RNA was isolated using the RNeasy Mini Kit (Qiagen). DNA contamination was removed using the TURBO DNA*-free* kit (Invitrogen), per manufacturer instructions, and confirmed by PCR amplification of the *flaB* gene (primers P411 and P412 in **Table S2**). cDNA for all samples was synthesized using the iScript cDNA Synthesis kit (Bio-Rad Laboratories) and assayed in three technical replicates for transcript levels of target genes quantified using the ddPCR system described above. Primers and probes are shown in **Table S2**. Samples were normalized to *flaB*. Values were expressed as an average ± standard error mean.

### Infection, Recovery, and Quantification of *B. burgdorferi* From Mice

Mammalian infectivity was assessed similarly to previous studies (Casselli et al., 2019). Four- to six-week-old, male immunocompetent C3H/HeJ mice (C3H; Jackson, Sacramento, CA) were infected by needle inoculation in the subcutis over the shoulder blades with 100 ul of BSK-II containing 1 x 10^2^, 1 x 10^3^, and 1 x 10^4^ *B. burgdorferi* cells. Three mice were used per group.

For spirochete detection by culture, blood, ear, heart, urinary bladder, and tibiotarsal joint were sampled at indicated time points and cultured in BSK-II supplemented with 20μg/ml phosphomycin, 50 μg/ml rifampicin, and 2.5 μg/ml amphotericin B. The presence or absence of spirochetes were assessed by dark-field microscopy for 4 weeks. The 50% infectious dose (ID50) was calculated using the Reed-Muench method (Reed and Muench, 1938).

For bacterial burden quantification, mice were infected with 1 x 10^4^ *B. burgdorferi* for 3 weeks and then humanely euthanized. Skin from the inoculation site, heart, and urinary bladder were snap-frozen in liquid nitrogen and stored at -80°C. DNA was extracted using the DNeasy Blood and Tissue kit (Qiagen) and then purified with the Genomic DNA Clean and Concentrator-10 kit (Zymo Research), both per manufacturer’s instructions. Three technical replicates were performed for each of the three biological replicates. qPCR for *B. burgdorferi flaB* and mouse *β-actin* was performed as described above with an annealing and extension temperature of 58°C. Relative copies of *flaB* per mouse *actin* were expressed as an average ± standard error mean.

### Statistics

For the cell density and qPCR data over the growth curve, comparisons were performed using a paired, two-tailed Student’s *t*-test was performed. For all other qPCR and qRT-PCR analyses, comparisons were made using an unpaired, two-tailed Student’s *t*-test. P < 0.05 was considered significant unless otherwise noted.

## Results

### Generation and Characterization of Δ*21-22* and lp17comp *B. burgdorferi* Clones

**Figure 1:**
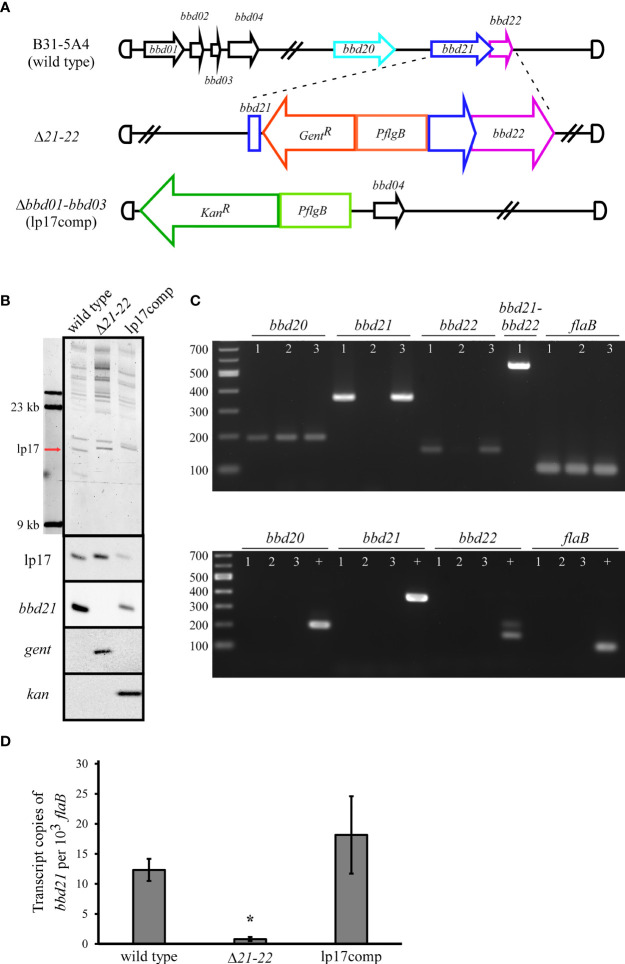
Characterization of the *bbd21-bbd22* mutant (Δ*21-22*) and complement (lp17comp). **(A)** Schematic of the *B. burgdorferi* strains generated in this study. The deletion mutant was generated in the 5A4 strain by allelic exchange resulting in a targeted internal deletion within *bbd21* and replacement by a *gentamicin* resistance cassette driven by a *flgB* promoter. The lp17comp clone was generated by whole plasmid transformation of a 5A4 mutant in which *bbd01-bbd03* were replaced by a *kanamycin* resistance cassette driven by *flgB*. **(B)** The field inversion gel electrophoresis demonstrates 1 µg of genomic DNA loaded per lane, 23 kb and 9.4 kb size molecular markers, and the location of the lp17 plasmid (red arrow). The expected increased plasmid size of the deletion mutant and complement strains result from addition of antibiotic resistance markers. Southern blot analyses of wild-type, Δ*21-22*, and lp17comp genomic DNA probed for lp17, *bbd21*, *kanamycin*, and *gentamicin*. **(C)** The upper gel depicts reverse-transcription PCR assessment of *bbd21* deletion. Total RNA was isolated, converted to complementary DNA, and used to amplify regions within *bbd21*, the flanking genes *bbd20* and *bbd22*, an overlapping region of *bbd21-bbd22*, and *flaB*. 1 = wild type, 2 = Δ*21-22*, and 3 = lp17comp. A 194 bp region of *bbd20* was amplified using P1399 and P1400, a 362 bp region within the *bbd21* internal deletion was amplified using P1056 and P1057, a 157 bp region of *bbd22* was amplified using P1401 and P934, a 565 bp region overlapping *bbd21* and *bbd22* was amplified using P1299 and P934, and a 105 bp region of *flaB* was amplified using P411 and P412. The lower gel depicts RNA template (no transcriptase) negative controls and wild-type genomic DNA positive controls (+). **(D)** qRT-PCR analyses of *bbd21* transcription from *in vitro*-cultivated wild-type, Δ*21-22*, and lp17comp strains. Transcript levels for *bbd21* were normalized to 10^3^ copies of *flaB* using a TaqMan-assay. Values were expressed as an average of three experiments ± standard error mean. *p<0.05 (Student’s *t*-test).

The *bbd21* gene was proposed as a potential *parA* orthologue involved with plasmid maintenance (Zückert and Meyer, 1996; Casjens et al., 2000). However, targeted deletion of the individual *bbd21* gene and detailed, quantitative analyses on its impact have not been performed to date. To investigate the function of *bbd21*, a deletion mutant and its complement were generated in the B31-5A4 background (**Figure 1A**). A detailed schematic including primer and probe descriptions is presented in **Supplementary Figure 1**. Briefly, the deletion mutant (Δ*21-22*) was made by insertion of a *gentamicin* resistance cassette into the *bbd21* ORF through allelic exchange. After numerous unsuccessful attempts to complement *bbd21* into its native locus in the deletion mutant, a previously published alternative strategy using whole plasmid replacement (Grimm et al., 2004; Imai et al., 2013; Raffel et al., 2014) was utilized to generate the complement strain, lp17comp. The lp17comp strain was generated by whole plasmid transformation of a previously published lp17 mutant in which *bbd01-bbd03* were replaced by a *kanamycin* resistance marker, and the mutant had no reported effect on murine infectivity and tissue colonization (Tourand et al., 2003; Casselli et al., 2019). Field inversion gel electrophoresis and Southern blot analyses confirmed successful genetic manipulation of the mutant strains (**Figure 1B**). The field inversion gel demonstrates 1 µg of genomic DNA loaded per lane, 23 kb and 9.4 kb size molecular markers, and the location of the lp17 plasmid. The size of lp17 for wild type is 16,823 bp. For Δ*21-22*, the 408 bp internal deletion of *bbd21* and the addition of 988 bp for the *flgB* promoter and *gentamicin* resistance cassette resulted in a size of 17,403 bp for lp17. For lp17comp, the 1395 bp deletion of *bbd01-bbd03* and addition of 2608 for the *flgB* promoter, *kanamycin* resistance cassette, and part of the plasmid vector resulted in a size of 18,036 for lp17. Southern blot analyses confirmed maintenance of lp17 in all strains by detection of *bbd12-bbd13* near the plasmid replication initiator; the absence of *bbd21* in the deletion mutant and restoration in the complement; and the presence of the appropriate antibiotic resistance markers in the respective mutants. Additionally, PCR amplification of the region flanking the internal *bbd21* deletion supported successful insertion of the antibiotic resistance gene into *bbd21* and restoration of the gene in the complement (**Supplementary Figure 2**). RT-PCR analyses revealed that *bbd21* deletion resulted in loss of *bbd21* and *bbd22* transcription, and that a single transcript from overlapping regions of these genes indicated co-transcription (**Figure 1C**). Significantly decreased transcription of *in vitro bbd21* corroborated disruption of the gene in the deletion mutant (**Figure 1D**) and demonstrated restoration of wild-type levels in the complement clone. The endogenous plasmid profiles of both mutants were similar to the wild type except for the loss of cp9 (data not shown), a plasmid that is often lost in culture and considered nonessential for mouse infectivity (Golde and Dolan, 1995; Purser and Norris, 2000; Labandeira-Rey and Skare, 2001; McDowell et al., 2001).

**Figure 2:**
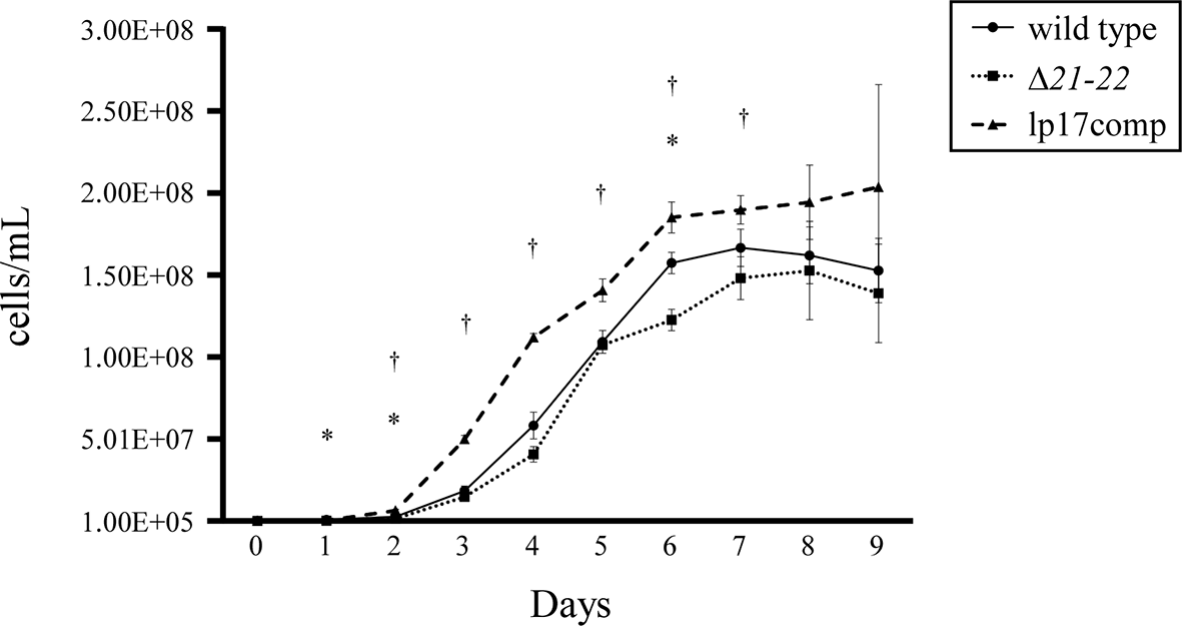
Deletion of *bbd21-bbd22* leads to decreased growth rate *in vitro*. At 24-h intervals, data points represent the average of cells/ml from three experiments, and error bars represent standard deviation. *wild type vs. Δ*21-22*, ^†^wild type vs. lp17comp; p<0.05 (Student’s *t*-test).

It has been previously reported that *B. burgdorferi* lacking *bbd16* to *bbd25* had impaired growth throughout the entirety of *in vitro* cultivation (Casselli et al., 2012). To assess the impact of *bbd21-bbd22* deletion on growth, wild-type, Δ*21-22*, and lp17comp strains were grown in liquid BSK-II, and averaged densities were compared every 24 h of the growth curve. The mutant clones showed altered growth compared to the wild type (**Figure 2**). Average bacterial densities at each time point are listed in **Supplementary Table 3**. The Δ*21-22* strain displayed a significantly delayed early growth on days 1 and 2 as well as decreased spirochetes in the early stationary phase on day 6. Conversely, lp17comp displayed significantly increased growth density compared to wild type during exponential growth from days 2 – 7. Overall, the altered growth exhibited by these mutants indicate *bbd21* and *bbd22* affect spirochete replication.

### Deletion of *Bbd21*-*bbd22* Results in Dysregulated Lp17 Copy Number Following *In Vitro* Passaging

**Figure 3:**
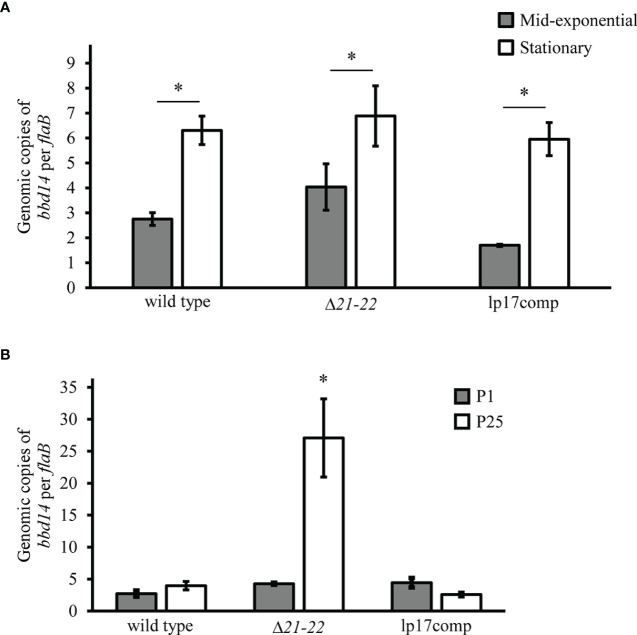
Deletion of *bbd21-bbd22* results in changes to lp17 plasmid copy number at different stages of the growth curve and following *in vitro* passaging. **(A)** Quantitative PCR analysis of genomic DNA from wild-type, Δ*21-22*, and lp17comp strains grown to mid-exponential phase (~2 x 10^7^ cells/ml; gray bars) and stationary phase (~1.2 x 10^8^ cells/ml; white bars). Bars indicate the average relative copies of *bbd14* per *flaB* from three experiments ± standard error mean. *p<0.05 (Student’s *t*-test). **(B)** *in vitro* Quantitative PCR of genomic DNA isolated from bacterial strains sepassaged 25 times *in vitro*. Three samples per strain were used, and values were expressed as averaged copies of *bbd14* per *flaB* ± standard error mean. Samples passaged 25 times (P25) are displayed in white bars, and those cultivated from glycerol stocks (P1) not passaged more than three times prior to the start of experiments are displayed as gray bars. Error bars represent standard error mean. *p<0.05 (Student’s *t*-test).

Previous studies have suggested that *bbd21* is involved with lp17 plasmid replication based on physical interaction of recombinant BBD21 with BBD14 (the protein product of the gene required for lp17 replication) during purification, although this was not confirmed in additional experiments by the same authors (Deneke and Chaconas, 2008). To further investigate the potential role of *bbd21* on lp17 copy number, plasmid copies were evaluated over the growth curve and over multiple generations. While there were significantly increased lp17 copies for each strain in the stationary phase (~1.2 x 10^8^ cells/ml) compared to mid-exponential phase (~2 x 10^7^ cells/ml), there was no difference among strains at either growth phase (**Figure 3A**). To investigate the effect of *bbd21-bbd22* deletion over multiple generations, qPCR probing for *bbd14* was performed on genomic DNA to quantify the number of lp17 plasmids in each *B. burgdorferi* strain following 25 *in vitro* passages. Results showed a significant ~6-8-fold increase in lp17 copy number by qPCR in passaged Δ*21-22* spirochetes compared to wild type and lp17comp (**Figure 3B**). These results indicate the *bbd21*-*bbd22* region is involved with lp17 plasmid copy number after passaging.

### *Bbd21* Transcription and Lp17 Copy Number Differ Between *In Vitro*- and DMC-Cultivated Spirochetes

**Figure 4:**
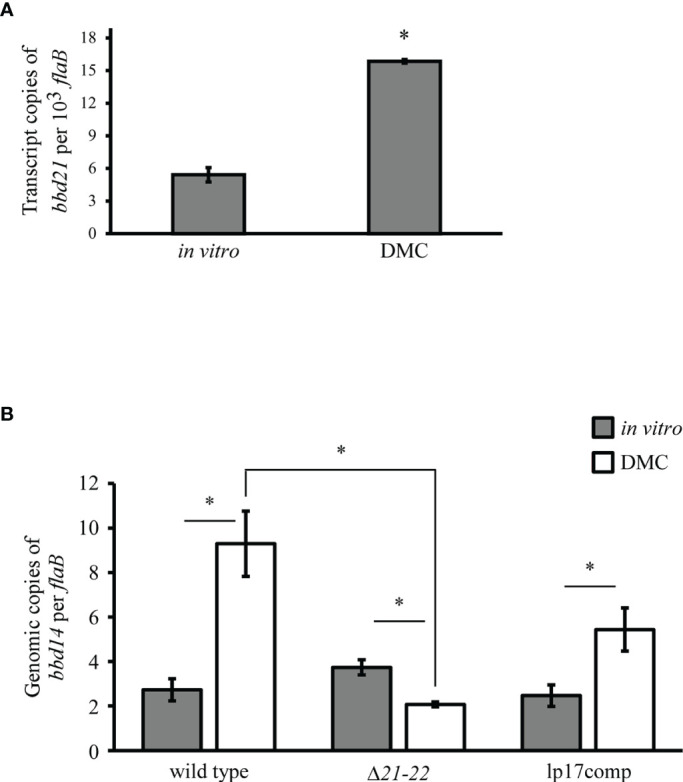
*bbd21* transcription and lp17 copy number are expressed differently in DMC-cultivated vs. *in vitro*-cultivated spirochetes. Mammalian host-adapted spirochetes were generated in dialysis membrane chambers (DMCs) implanted into the peritoneal cavity of rats. **(A)** qRT-PCR analysis of *bbd21* expression in *in vitro*- vs. DMC-cultivated wild type spirochetes. Bars represent the mean values of three independent experiments, and error bars represent standard error mean. *p<0.05 (Student’s *t*-test). **(B)** qPCR analyses of lp17 copy number in wild-type, Δ*21-22*, and lp17comp strains. *In vitro* conditions are depicted as gray bars, and DMC conditions are depicted as white bars. The bars represent the mean values of three independent experiments, except in the Δ*21-22* DMC-cultivated spirochetes in which one animal died during surgery (n=2). The error bars represent standard error mean. *p<0.05 (Student’s *t*-test).

The *B. burgdorferi* 5A4 strain used in this study was isolated from a tick host (Burgdorfer et al., 1982). It is well-known that the spirochete undergoes drastic antigenic profile changes in response to host environmental signals. While differential gene expression between tick and mammalian host environments is well-studied in *B. burgdorferi*, differential endogenous plasmid copy number has not been investigated. To explore the role of *bbd21-bbd22* in mammalian host-adapted spirochetes, bacteria were cultivated using the dialysis membrane chamber (DMC) model (Caimano, 2018). First, to establish if *bbd21* is upregulated or downregulated in the mammalian host, transcript levels in wild-type spirochetes from *in vitro*- and DMC-cultivated spirochetes were assessed. Expression of *bbd21* was significantly increased 3-fold in DMCs compared to *in vitro* (**Figure 4A**). This upregulation indicates *bbd21* is induced during mammalian host adaptation.

Because *bbd21* was induced during mammalian host adaptation, the impact on lp17 copy number was evaluated between *in vitro* and DMC cultivation. By qPCR, all strains displayed significantly different lp17 copy number between *in vitro* and DMC conditions. Wild-type spirochetes grown *in vitro* harbored 3.4-fold fewer copies of lp17 compared to spirochetes grown in DMCs (**Figure 4B**). The inverse was observed between Δ*21-22* spirochetes grown *in vitro* and in DMCs. For lp17comp, lp17 copy number was 2.2-fold decreased under *in vitro* conditions compared to DMC. *in vitro*. While there was no statistical difference among strains grown under *in vitro* conditions, differences were observed under DMC conditions. The Δ*21-22* spirochetes contained significantly fewer copies of lp17 by 4.5-fold compared to wild-type. The mutant also contained 2.6-fold fewer copies of lp17 compared to lp17comp, although this was not statistically significant. Together, these results suggest that lp17 plasmid copy number is impacted by *in vitro* versus DMC growth conditions and by the expression of *bbd21-bbd22*.

### Deletion of *Bbd21-Bbd22* Results in Impaired Mouse Heart Tissue Colonization

Given the difference in lp17 copy number observed in DMCs by the Δ*21-22* strain combined with the recognition of lp17 as a plasmid encoding virulence factors important for mammalian adaptation (Sarkar et al., 2011; Casselli et al., 2012; Casselli et al., 2019; Medina-Pérez et al., 2020), mouse infection studies were performed to determine if the lack of *bbd21-bbd22* influences the ability of *B. burgdorferi* to colonize mammalian tissues. C3H mice were infected, and samples of blood, ear, heart, urinary bladder, and tibiotarsal joint were collected for culture. Infection rates were dose dependent for all strains, and the ID_50_ for Δ*21-22* was at least 10-fold higher than wild type for all tissues (**Table 1**).

**Table 1:** Minimum infectious dose and tissue colonization of wild-type, Δ*21-22*, and lp17comp *B. burgdorferi* strains in immunocompetent C3H mice.

Challenge dose	Tissue (dpi)	Challenge strain		
		wild type	Δ*21-22*	lp17comp
		No. culture-positive/total no. samples		
10^4^	Blood (7)	3/3	3/3	3/3
10^3^		2/3	0/3	2/3
10^2^		1/3	0/3	0/3
ID50		316	3162	558
10^4^	Ear (28)	3/3	2/3	3/3
10^3^		2/3	0/3	2/3
10^2^		1/3	0/3	0/3
ID50		316	5575	558
10^4^	Heart (28)	3/3	0/3	3/3
10^3^		2/3	0/3	2/3
10^2^		1/3	0/3	0/3
ID50		316	ND	558
10^4^	Bladder (28)	3/3	2/3	3/3
10^3^		2/3	0/3	2/3
10^2^		1/3	0/3	0/3
ID50		316	5575	558
10^4^	Joint (28)	3/3	2/3	3/3
10^3^		2/3	0/3	2/3
10^2^		1/3	0/3	0/3
ID50		316	5575	558

dpi = days post infection.

ND = not determined.

**Figure 5:**
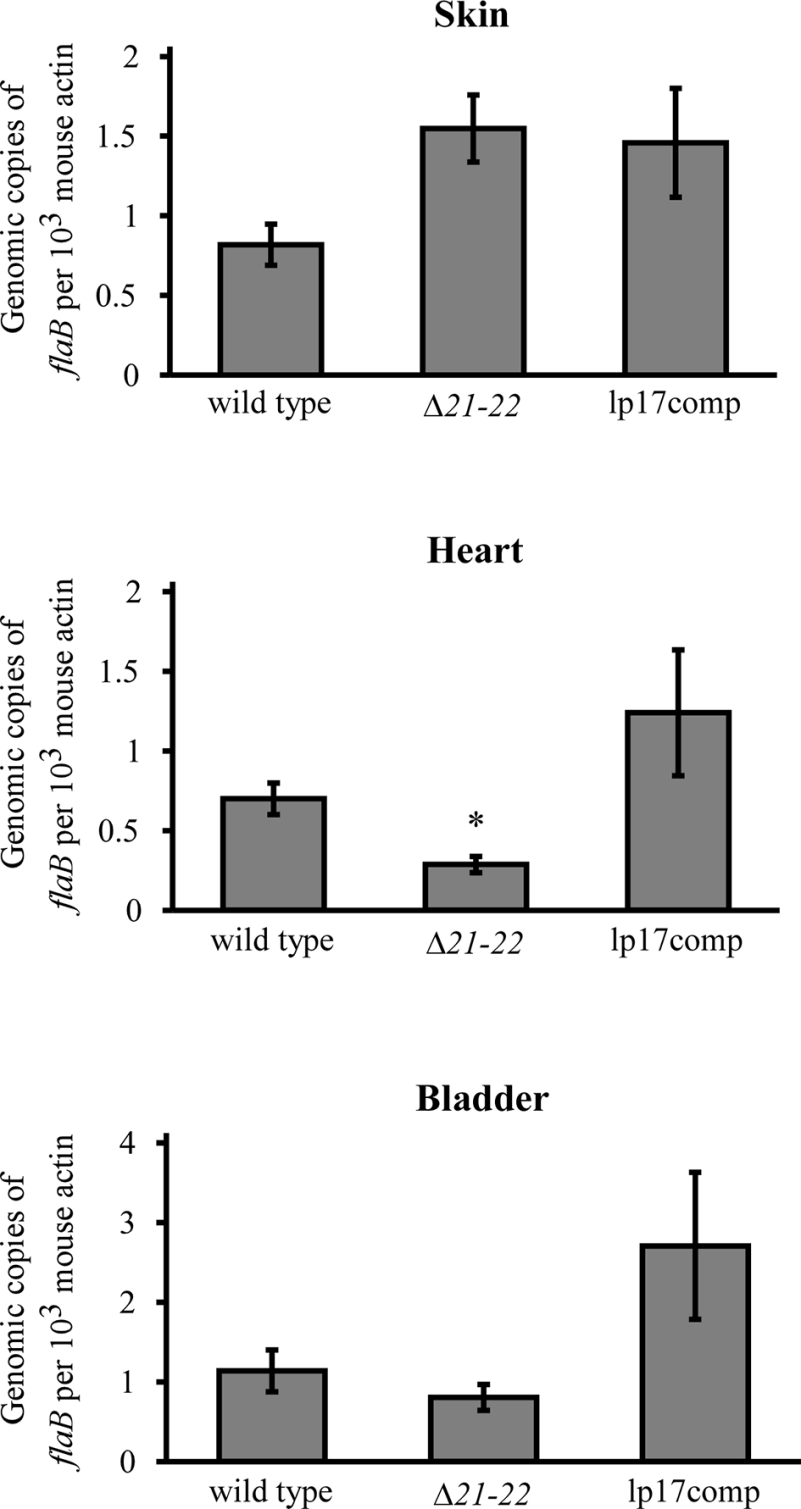
*bbd21-bbd22* is required for efficient colonization of the heart in immunocompetent mice. Three C3H mice per group were infected in the subcutis over the dorsum with 1x10^4^ spirochetes. Skin at the inoculation site, heart, and urinary bladder were collected at 21 days post-infection. Spirochete burden was assessed by qPCR analysis of genomic DNA isolated from these tissues. Gray bars represent the mean values of **(*B*)** *burgdorferi flaB* copies normalized to 10^3^ mouse *actin* copies, and error bars represent standard error mean. *p<0.05 (Student’s *t*-test).

In addition to culture, qPCR was performed to quantify spirochete burden. A mouse infection study was performed identically to the infection and spirochete recovery experiments at the 10^4^ inoculation dose. Skin at the inoculation site, heart, and bladder were harvested, and genomic DNA was analyzed by qPCR. Consistent with the culture results, spirochete burden in the heart was significantly decreased 2.4-fold for Δ*21-22* compared to wild type and lp17comp (**Figure 5**). For the dermal inoculation site, Δ*21-22* and lp17comp had mildly increased burden compared to wild type. For the bladder, lp17comp was approximately twice the burden of wild type and Δ*21-22*, although neither the skin nor bladder exhibited statistically significant differences. Altogether, these culture and qPCR results indicate that *bbd21* is involved with mouse tissue dissemination or colonization, particularly in the heart.

### Expression of *Bbd21* Is Independent of *RpoS*

**Figure 6:**
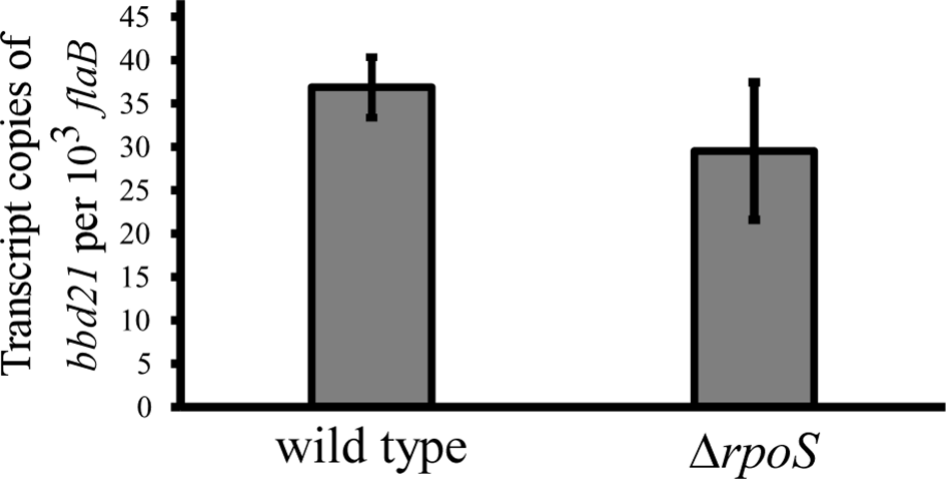
*bbd21* transcription is independent of *rpoS* regulation. Mammalian host-adapted spirochetes were generated in dialysis DMCs implanted into the peritoneal cavity of rats. Transcription of *bbd21* was compared by qRT-PCR analysis of wild-type and Δ*rpoS* spirochetes in three independent experiments. Gray bars represent mean values of *bbd21* transcript copies normalized to 10^3^ *flaB*, and error bars represent standard error mean. There is no statistical difference between the two strains (Student’s *t*-test).

The current study found that *bbd21* transcription was induced in mammalian host-adapted spirochetes. Additionally, lp17 copy number was observed to be altered in the deletion mutant in this environment. This begs the question if *bbd21* is regulated by the canonical alternative σ factor cascade. The BosR/Rrp2/RpoN/RpoS alternative σ factor cascade is the most well-studied global regulator of *B. burgdorferi* gene expression throughout the enzootic life cycle (Hubner et al., 2001; Yang et al., 2003; Caimano et al., 2007; Hyde et al., 2009; Ouyang et al., 2009; Ouyang et al., 2011; Groshong et al., 2012; Ouyang et al., 2014; Caimano et al., 2019). Despite the global regulation this network displays, neither *bbd21* nor *bbd22* is reported to be impacted by mutations to this pathway in microarray experiments (Caimano et al., 2007; Ouyang et al., 2009; Caimano et al., 2019). To investigate the potential role of *rpoS* regulation on *bbd21*, wild type and Δ*rpoS* spirochetes were cultivated in DMCs and evaluated for *bbd21* transcript levels by qRT-PCR (**Figure 6**). The results from qRT-PCR analysis showed that there was no statistical significance between expression levels of the two strains, which further suggests that *rpoS* does not regulate *bbd21* expression.

## Discussion

Our findings reveal that a role of the *B. burgdorferi* gene products of the *bbd21-bbd22* genetic region is lp17 plasmid copy number regulation. We demonstrated that deletion of *bbd21-bbd22* expression resulted in a significant increase of lp17 copy number following *in vitro* passaging, and that complementation returned plasmid copy number to wild-type levels. Copy number of lp17 was previously reported to be one copy per chromosome by DNA hybridization (Hinnebusch and Barbour, 1992), which was limited by the sensitivity of electrophoretic analysis. By qPCR, a previous study determined lp17 copy number to be ~3.1 per the chromosome end and 0.8 per the chromosome center, with the discrepancy attributed to multiple replication centers and initiation events on the chromosome (Beaurepaire and Chaconas, 2007). In the current study, wild-type and lp17comp spirochetes displayed similar lp17 copies per chromosome end ranging from 2.3 to 5.2. Additionally, an earlier study speculated that the absence of PF32 would result in loss of plasmids during *in vitro* cultivation (Zückert and Meyer, 1996). On the contrary, the present study revealed an 8-fold increase of lp17 copies following *bbd21-bbd22* deletion following *in vitro* passaging. The drastic increase observed in lp17 copy number by the Δ*21-22* mutant was not observed in previous reports involving *bbd21* deletion (Beaurepaire and Chaconas, 2005; Casselli et al., 2012), which is likely due to conducting multiple passages coupled with quantitative analysis in the present study.

While our findings suggest that the *bbd21-bbd22* genetic region is involved with plasmid copy number regulation, the precise function (i.e., plasmid replication, plasmid partitioning, or plasmid copy number control) remains obscure. The finding of increased plasmid copy number following *in vitro* passaging in Δ*21-22* spirochetes may suggest that *bbd21* functions less as a strict ParA partitioning protein and more like the RepB regulator of plasmid copy number in *Enterococcus faecalis* (Weaver et al., 1993). RepB is a member of the ParA superfamily of ATPases and contains sequence identity with *bbd21* (Zückert and Meyer, 1996). In *E. faecalis*, RepB functions with RepC, the ParB orthologue, to stabilize the plasmid partitioning complex (Francia et al., 2007). However, a RepC/ParB orthologue has yet to be identified in the *B. burgdorferi* genome. Furthermore, the finding of significantly decreased lp17 copy number in DMC-cultivated spirochetes lacking *bbd21-bbd22* expression contrasts the *in vitro* passaging results and may suggest *bbd21-bbd22* serves a distinct function for plasmid copy number regulation during mammalian adaptation.

Several reports have speculated that PF members may act *in trans* to supply gene products required for plasmid maintenance, replication, or partition to another plasmid that is lacking such factors (Eggers et al., 2002; Stewart et al., 2003; Beaurepaire and Chaconas, 2005; Beaurepaire and Chaconas, 2007; Tilly et al., 2012; Chaconas and Norris, 2013). In the current study, the deletion of *bbd21-bbd22* and subsequent increase in lp17 copy number following *in vitro* passaging and decrease in lp17 copy number in mammalian host-adapted spirochetes suggests that this PF32 member is not compensated *in trans* by PF32 members encoded on other plasmids in the *B. burgdorferi* genome. Future studies are warranted to investigate if *bbd21-bbd22* may impact copy number of other endogenous plasmids as well.

In addition, we demonstrated that *B. burgdorferi* strains can harbor significantly different copies of lp17 under *in vitro* versus mammalian host-adapted conditions. This suggests that mammalian host factors not only influence transcriptome expression, but also *bbd21-bbd22* and endogenous plasmid copy number. This difference in copy number raises the possibility of gene dose-associated expression. That is, with differing copy numbers of lp17 due to *in vitro* versus DMC cultivation or *bbd21-bbd22* deletion, there may be altered expression of all genes encoded on the plasmid based on copy number variation. Given that lp17 encodes factors that regulate virulence (Sarkar et al., 2011; Casselli et al., 2012; Casselli et al., 2019; Medina-Pérez et al., 2020), as well as genes of unknown function, the impact on the transcriptome is difficult to predict. Nonetheless, a practical implication of this study is that it highlights the need for copy number analysis in mutagenesis studies, particularly if a region near a PF32 member may be affected.

In the mouse model, deletion of *bbd21-bbd22* resulted in decreased infectivity based on culture results in all tissues, increased ID_50_, and spirochete burden in murine heart tissue. We postulate that the infectivity defect could be associated with gene dosage and dysregulated expression of virulence factors. It has been described that insufficient expression or overexpression of outer surface proteins such as OspC, which is regulated by *bbd18* encoded on lp17, can lead to clearance by the host immune system (Tilly et al., 2006; Xu et al., 2006; Casselli et al., 2012). Similarly, it has been reported in other bacteria, such as *Yersinia pseudotuberculosis*, that upregulation of copy number for plasmids encoding key virulence factors is essential to establishing infection in a host (Wang et al., 2016). Thus, in the current study, it is possible that the attenuated murine tissue colonization may be associated with the significantly decreased lp17 copies observed in mammalian host-adapted spirochetes lacking *bbd21-bbd22*. Alternatively, the attenuated tissue colonization may be associated with the early growth defect which could result in partial clearance and decreased spirochete burden. Additional data is needed to support a possible link between plasmid copy number and pathogenicity in *B. burgdorferi*.

Expression of *bbd21* does not appear to be governed by *rpoS*. Comparison of *bbd21* transcript levels between *in vitro* grown wild-type and Δ*rpoS* spirochetes revealed no significant difference, suggesting *bbd21* expression is independent of the gatekeeper pathway. This is consistent with the absence of *bbd21* from transcriptomic studies involving disruption of the alternative σ factor cascade (Caimano et al., 2007; Ouyang et al., 2009; Caimano et al., 2019). One possible explanation is that several plasmid partitioning and replication proteins are known to negatively self-regulate (Funnell and Slavcev, 2014). Alternatively, *bbd21* may be regulated in association with the stringent response, as expression of the gene is significantly increased in a microarray study of a DksA-deficient strain during nutrient stress (Boyle et al., 2019). Furthermore, both *bbd21* and *bbd22* were identified in transcriptomic experiments as upregulated genes in starvation-adapted cells starved for N-acetylglucosamine (Schneider and Rhodes, 2018). Given the increased metabolic requirement to fuel plasmid number expansion during replication, plasmid copy number dysregulation may account for the delayed growth densities observed for the Δ*21-22* mutant in the current study. Current studies are underway to determine a possible link between *bbd21-bbd22* plasmid copy regulation and metabolism.

Because *bbd21* was previously proposed as a putative *parA* orthologue based on 25% sequence homology and presence of a Walker box motif (Zückert and Meyer, 1996), as well as demonstrated ATPase activity (Deneke and Chaconas, 2008), it seems plausible that the deletion of this gene is responsible for the changes in lp17 plasmid copy number. However, our findings revealed that *bbd21* and *bbd22* are co-transcribed. Therefore, contribution to plasmid copy number regulation from *bbd22* cannot be definitively ruled out in this study. Further investigation of *bbd22* is required to determine its potential role in plasmid copy number regulation, particularly as identification of a *parB*/*repC* orthologue remains elusive in the *B. burgdorferi* genome. Alternatively, *bbd22* may not be involved with plasmid copy number and instead may serve a function not directly observed in this study.

In conclusion, our study describes a role of the *bbd21-bbd22* genetic region that involves lp17 plasmid copy number regulation and raises the question of gene dosage in Lyme disease pathogenesis. Furthermore, the results described herein add to the growing body of work implicating the importance of lp17, and highlight the need for additional studies aimed at elucidating the mechanisms of *bbd21-bbd22* plasmid copy control and how this putative bicistronic operon is regulated in this important pathogen.

## Data Availability Statement

The original contributions presented in the study are included in the article/**Supplementary Material**. Further inquiries can be directed to the corresponding author.

## Ethics Statement

The animal study was reviewed and approved by Washington State University Institutional Animal Care and Use Committee.

## Author Contributions

JW and TB designed the experiments. JW and MC performed the experiments. JW and TB analyzed the data and wrote the manuscript. All authors contributed to the article and approved the submitted version.

## Funding

This work was supported by an Intramural grant from the College of Veterinary Medicine at Washington State University (TB), and a Poncin fellowship (JW).

## Conflict of Interest

The authors declare that the research was conducted in the absence of any commercial or financial relationships that could be construed as a potential conflict of interest.

## Publisher’s Note

All claims expressed in this article are solely those of the authors and do not necessarily represent those of their affiliated organizations, or those of the publisher, the editors and the reviewers. Any product that may be evaluated in this article, or claim that may be made by its manufacturer, is not guaranteed or endorsed by the publisher.
