# Supplementary material for: Deletion of a Genetic Region of lp17 Affects Plasmid Copy Number in Borrelia burgdorferi
Source: Front Cell Infect Microbiol. 2022 Apr 12;12:884171. doi: 10.3389/fcimb.2022.884171 (PMC9039534; doi:10.3389/fcimb.2022.884171)
Supplement: Supplementary file 1 [file DataSheet_1.docx]

**Supplementary Table 1. Strains used in this study.**

| **Strain** | **Description** | **Reference** |
| --- | --- | --- |
| Wild type | *B. burgdorferi* strain B31 clone 5A4 | Purser and Norris, 2000 |
| Δ*21-22* | 5A4 strain lacking *bbd21* and *bbd22* expression | This Study |
| lp17comp | Δ*21-22* complemented with whole plasmid transformation of lp17Δ*bbd01-bbd03* | This Study |
| Δ*rpoS* | B31 clone 5A4 Δ*rpoS* mutant | Caimano et al., 2019 |

**Supplementary Table 2. Oligonucleotide primers used in this study.**

| **Primer Name** | **Sequence (5'-3')** | **Application** | **Reference** |  |  |
| --- | --- | --- | --- | --- | --- |
| P933 | CGTAGAATGAGACAAAAGCCGTGAATATAG | PCR amplification of *bbd21* and 305 bp upstream | This study |  |  |
| P934 | CAGTCCTACACTTATTTGCTTGATCG | PCR amplification of *bbd21* and 149 bp downstream; *bbd22* RT-PCR | This study |  |  |
| P996 | CATGATTTAATTAAATTTCCGCTTAAGGTAGTTTTCC | PCR amplification generating 408 bp internal deletion in *bbd21* with PacI restriction site | This study |  |  |
| P997 | CATGATACCGGTATTTTTAAAGAAGAGTTTGAGTCTTTG | PCR amplification generating 408 bp internal deletion in *bbd21* with AgeI restriction site | This study |  |  |
| P998 | CATGATACCGGTCAGTTGCGCAGCCTG AATGG | PCR amplification of *flgB*-*gent* with AgeI restriction site | This study |  |  |
| P508 | GCATTAATTAAAGGTGGCGGTACTTGGGTCG | PCR amplification of *flgB*-*gent* with PacI restriction site | Rogovskyy and Bankhead, 2014 |  |  |
| P172 | ACTGCAATCTGCCCAAGCTACATAATCT | *bbd12-bbd13* Southern blot | Purser and Norris, 2000 |  |  |
| P173 | AAGGTAAGGACGGTTGTCTACATGGATT | *bbd12-bbd13* Southern blot | Purser and Norris, 2000 |  |  |
| P1056 | GTTGATTGTGATATACAGCAAGC | *bbd21* internal deletion RT-PCR/Southern blot | This study |  |  |
| P1057 | CCTTCAAGGCTTAAAAATTCTGGG | *bbd21* internal deletion RT-PCR/Southern blot | This study |  |  |
| P91 | CGCAGCAGCAACGATGTTAC | *gentamicin* PCR/Southern blot | Tourand et al., 2006 |  |  |
| P92 | CTTGCACGTAGATCACATAAGC | *gentamicin* PCR/Southern blot | Tourand et al., 2006 |  |  |
| P54 | CATATGAGCCATATTCAACGGGAAACG | *kanamycin* PCR/Southern blot | Bono et al., 2000 |  |  |
| P55 | AAAGCCGTTTCTGTAATGAAGGAG | *kanamycin* PCR/Southern blot | Bono et al., 2000 |  |  |
| P411 | GAGTTTCTGGTAAGATTAATGCTC | *flab* PCR | Magunda and Bankhead, 2016 |  |  |
| P412 | CATTTAAATTCCCTTCTGTTGTCTGA | *flab* PCR | Magunda and Bankhead, 2016 |  |  |
| P1399 | GTGTTAAAGCGGCAAGATAG | *bbd20* RT-PCR | This study |  |  |
| P1400 | GAAATCCTTGTGTTCTATCCCTC | *bbd20* RT-PCR | This study |  |  |
| P1401 | GCCTATAAGCAAAGAGGTTAATTTAGAAG | *bbd22* RT-PCR | This study |  |  |
| P1365 | CGCTCAAGGCTAAAATCATATTTAAC | *bbd14* qPCR | This study |  |  |
| P1366 | CTATTGTGGAATATGTTTTAAGTTTGTTAG | *bbd14* qPCR | This study |  |  |
| P1367 | HEX-CCAACGAATACTCAAAGTATGCTGGGC-BHQ1 | *bbd14* qPCR | This study |  |  |
| P199 | TTGCTGATCAAGCTCAATATAACCA | *flaB* qPCR, qRT-PCR | This study |  |  |
| P200 | TTGAGACCCTGAAAGTGATGC | *flaB* qPCR, qRT-PCR | This study |  |  |
| P201 | FAM-CAGCTGAAGAGCTTGGAATGCAGCCT-TAMRA | *flaB* qPCR, qRT-PCR | This study |  |  |
| P202 | AGAGGGAAATCGTGCGTGAC | mouse *actin* qPCR | This study |  |  |
| P203 | CAATAGTGATGACCTGGCCGT | mouse *actin* qPCR | This study |  |  |
| P1086 | HEX-CACTGCCGCATCCTCTTCCTCCC-BHQ1 | mouse *actin* qPCR | This study |  |  |
| P1299 | GTTTGGAATTGGAGAAATTGGG | *bbd21* qRT-PCR | This study |  |  |
| P1300 | CCTTCAAGGCTTAAAAATTCTGGG | *bbd21* qRT-PCR | This study |  |  |
| P1301 | HEX-CTTCGCTCCCAAAGCTCAAAACTGGG-BHQ1 | *bbd21* qRT-PCR | This study |  |  |
| Underline indicate restriction enzyme sites. | | | | |  |

**Supplementary Table 3. *In vitro* growth rates of *Borrelia* strains.** Data represents the average density of cells per ml from three experiments for each strain at 24-h intervals.

|  | Day 0 | Day 1 | Day 2 | Day 3 | Day 4 | Day 5 | Day 6 | Day 7 | Day 8 | Day 9 |
| --- | --- | --- | --- | --- | --- | --- | --- | --- | --- | --- |
| wild type | 1.0E+05 | 8.3E+05 | 2.7E+06 | 1.9E+07 | 5.8E+07 | 1.1E+08 | 1.6E+08 | 1.7E+08 | 1.6E+08 | 1.5E+08 |
| Δ*21-22* | 1.0E+05 | 2.4E+05 | 1.6E+06 | 1.5E+07 | 4.1E+07 | 1.1E+08 | 1.2E+08 | 1.5E+08 | 1.5E+08 | 1.4E+08 |
| lp17comp | 1.0E+05 | 5.8E+05 | 6.5E+06 | 5.0E+07 | 1.1E+08 | 1.4E+08 | 1.9E+08 | 1.9E+08 | 1.9E+08 | 2.0E+08 |

**Supplementary Figure 1. Detailed schematic of Δ2*1-22* and lp17comp with indicated primers and probes.** Amplification of P933 and P934 yields a 1195 bp amplicon in wild type and lp17comp. The same primers yield a 1775 bp amplicon in Δ2*1-22* due to addition of the *flgB* promoter and gentamicin resistance gene. Primers P998 and P508 were used to clone in a 992 bp *flgB* promoter and *gentamicin* resistance gene into the deletion construct. Digoxigenin-labeled DNA probes were synthesized using P172 and 173 for *bbd12-bbd13* near the plasmid replication center, P1056 and P1057 for the internal deletion of *bbd21*, P91 and P92 for the gentamicin resistance gene, and P54 and P55 for the kanamycin resistance gene. For RT-PCR, a 194 bp region of *bbd20* was amplified with P1399 and P1400, a 362 bp region within the *bbd21* internal deletion was amplified with P1056 and P1057, and a 157 bp region of *bbd22* was amplified with P1401 and P934. A 105 bp region of *flaB* was amplified with P411 and P412 (not pictured). Relative lp17 plasmid copy number was identified by qPCR of the plasmid replication center, *bbd14*, using P1365 and P1366. Transcription of *bbd21* was evaluated by qRT-PCR using P1299 and P1300.

**Supplementary Figure 2. PCR assessment of the *bbd21* region following genetic manipulation.** Genomic DNA from bacterial strains was amplified using primers that flank the *bbd21* internal deletion (P933 and P934). Wild type and lp17comp display similarly sized 1195 bp amplicons. The Δ*21-22* exhibits the expected band size of 1775 bp due to the 408 bp *bbd21* internal deletion and addition of 988 bp from the *flgB* promoter and *gentamicin* resistance gene.

References

Bono, J. L., Elias, A. F., Kupko, J. J., Stevenson, B., Tilly, K., and Rosa, P. (2000). Efficient targeted mutagenesis in *Borrelia burgdorferi*. *J Bacteriol* 182, 2445–2452. doi:10.1128/JB.182.9.2445-2452.2000.

Caimano, M. J., Groshong, A. M., Belperron, A., Mao, J., Hawley, K. L., Luthra, A., et al. (2019). The RpoS Gatekeeper in *Borrelia burgdorferi*: An Invariant Regulatory Scheme That Promotes Spirochete Persistence in Reservoir Hosts and Niche Diversity. *Frontiers in Microbiology* 10. doi:10.3389/fmicb.2019.01923.

Magunda, P. R. H., and Bankhead, T. (2016). Investigating the potential role of non-*vls* genes on linear plasmid 28–1 in virulence and persistence by *Borrelia burgdorferi*. *BMC Microbiology* 16, 180. doi:10.1186/s12866-016-0806-4.

Purser, J. E., and Norris, S. J. (2000). Correlation between plasmid content and infectivity in *Borrelia burgdorferi*. *Proc Natl Acad Sci U S A* 97, 13865–70. doi:10.1073/pnas.97.25.13865.

Rogovskyy, A. S., and Bankhead, T. (2014). Bacterial heterogeneity is a requirement for host superinfection by the Lyme disease spirochete. *Infect Immun* 82, 4542–52. doi:10.1128/IAI.01817-14.

Tourand, Y., Bankhead, T., Wilson, S. L., Putteet-Driver, A. D., Barbour, A. G., Byram, R., et al. (2006). Differential Telomere Processing by *Borrelia* Telomere Resolvases *In Vitro* but Not *In Vivo*. *J Bacteriol* 188, 7378–7386. doi:10.1128/JB.00760-06.
